# Behavioral Activation for Depression and Glycemic Control in Type 2 Diabetes: A Systematic Review and Meta‐Analysis

**DOI:** 10.1002/brb3.71761

**Published:** 2026-09-15

**Authors:** Xiao Lu, Tian Xia, Zhuo Wang, Mengjiao Cheng, Rong Xu

**Affiliations:** ^1^ Department of Nursing Tongji Hospital, Tongji Medical College of Huazhong University of Science and Technology Wuhan China; ^2^ School of Nursing Tongji Medical College of Huazhong University of Science and Technology Wuhan China

**Keywords:** behavioral activation, meta‐analysis, systematic review, type 2 diabetes

## Abstract

**Introduction:**

Depression in type 2 diabetes mellitus (T2DM) hinders self‐management and glycemic control. Behavioral activation (BA), by increasing participation in positive activities, holds promise for simultaneously improving depressive symptoms and diabetes outcomes.

**Objective:**

This study evaluated the effects of BA or BA‐based interventions on depressive symptoms, glycated hemoglobin (HbA1c), diabetes self‐efficacy, and self‐management behaviors in adults with T2DM, and summarized their implementation characteristics.

**Methodology:**

PubMed, Web of Science, Embase, Cochrane Library, PsycINFO, CNKI, VIP, and Wanfang Data were searched from inception to February 1, 2026. Interventional studies assessing BA or BA‐based interventions in adults with T2DM and reporting at least one prespecified outcome were included. Quantitative meta‐analyses were restricted to randomized controlled trials (RCTs) and conducted using random‐effects models. Implementation characteristics from all included studies were narratively synthesized.

**Results:**

Six studies involving 252 participants met the inclusion criteria, including five RCTs and one single‐arm pre‐post study. The meta‐analysis suggested a reduction in depressive symptoms (SMD = −0.81, 95% CI: −1.22 to ‐0.41, *p* < 0.001), but no statistically significant effects on HbA1c (MD = −0.04%, 95% CI: −0.30 to 0.22, *p* = 0.76), diabetes self‐efficacy (SMD = 0.03, 95% CI: −0.30 to 0.35, *p* = 0.87), or self‐management behaviors (SMD = 0.04, 95% CI: −0.23 to 0.32, *p* = 0.77). Narrative synthesis indicated that BA interventions varied in delivery format, dosage, provider background, and integration with diabetes‐specific content. Most interventions were delivered by non‐mental health professionals and incorporated core BA components such as activity monitoring and goal setting.

**Conclusion:**

BA may reduce depressive symptoms in adults with T2DM, whereas its effects on glycemic control, diabetes self‐efficacy, and self‐management behaviors remain uncertain. BA has been delivered in flexible formats and may have potential for integration into diabetes care, but larger, well‐powered RCTs with longer follow‐up are needed to determine whether diabetes‐specific BA can improve behavioral and physiological outcomes.

## Graphical abstract

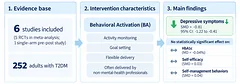

## 1 Introduction

Diabetes mellitus represents a major global public health challenge, with the International Diabetes Federation (IDF) estimating 589 million cases in 2024 (International Diabetes Federation n.d.), over 90% of which are type 2 diabetes mellitus (T2DM) (Zheng et al. 2018). Effective T2DM management requires sustained engagement in complex self‐management behaviors, including healthy diet, physical activity, medication adherence, and regular glycemic monitoring (Davis et al. 2022).

These continuous self‐management demands, coupled with the physiological stressors of the disease, substantially elevate the risk of depression. The prevalence of depression among individuals with T2DM is approximately double that observed in non‐diabetic populations, affecting nearly one in four adults worldwide (Roy and Lloyd 2012; Khaledi et al. 2019). Depressive symptoms, such as anhedonia, fatigue, and reduced motivation (American Psychiatric Association 2013), can impair treatment adherence and self‐management, thereby contributing to poorer glycemic control and reinforcing a negative cycle between psychological distress and diabetes outcomes (Gonzalez et al. 2008; Aikens 2012; Kamruzzaman et al. 2025).

Although various psychological interventions have been implemented, their reported effects on glycemic control remain inconsistent (Elliott 2012; van der Feltz‐Cornelis et al. 2021; Winkley et al. 2020). This inconsistency suggests that alleviating low mood alone may not be sufficient to promote the self‐management behaviors required for glycemic control. Behavioral activation (BA), a pragmatic behavioral therapy for depression, may be particularly relevant in this context because it focuses on increasing engagement in meaningful and value‐based activities (Hopko et al. 2003). Although its primary goal is to alleviate depression, the BA framework is well suited to the integration of self‐management goals: it allows patients to link difficult tasks, such as diet and exercise, with their core values, potentially helping patients reframe diabetes self‐management as a more meaningful and goal‐directed action.

In the broader literature on depression and diabetes care, BA or BA‐informed strategies have often been embedded within collaborative or integrated care models as one component of a multicomponent intervention. These models have shown benefits for depressive outcomes, and some evidence also suggests improvements in glycemic outcomes or medication adherence (Atlantis et al. 2014; Huang et al. 2013; Cooper et al. 2024). However, such models commonly combine BA with care management, medication management, self‐management support, and multidisciplinary follow‐up, making it difficult to isolate the specific contribution of BA (Schillok et al. 2025). In contrast, trials in which BA or BA‐based interventions serve as the primary or core therapeutic framework for adults with T2DM have reported mixed findings for glycated hemoglobin (HbA1c) and self‐management‐related outcomes (Ahmed et al. 2025; Bae et al. 2024).

Therefore, to our knowledge, no meta‐analysis has specifically synthesized evidence on BA as the primary or core intervention component in T2DM. This study aimed to quantitatively synthesize the effects of BA or BA‐based interventions on depressive symptoms, HbA1c, diabetes self‐efficacy, and self‐management behaviors in adults with T2DM. We also narratively synthesized the implementation characteristics of these interventions to inform future research and practice.

## 2 Methods

This systematic review and meta‐analysis was conducted and reported in accordance with the Preferred Reporting Items for Systematic Reviews and Meta‐Analyses (PRISMA) guidelines (Page et al. 2021). The review protocol is registered with the PROSPERO International Register of Systematic Reviews under the identifier CRD420251131642. A PRISMA checklist is available in Supplementary File Table S1.

### 2.1 Search Strategy

A systematic search of PubMed, Web of Science, Embase, Cochrane Library, PsycINFO, and three major Chinese databases (CNKI, VIP, and Wanfang Data) was performed from their inception up to February 1, 2026. A comprehensive search strategy, incorporating both subject headings and free keywords, was developed and implemented across these databases. Search terms included “Diabetes Mellitus, Type 2”, “T2DM”, “Type 2 Diabetes”, “behavioral activation”, “behavioral activation”, “behavior* activat*”, “behavior* activat*”, “activit* schedul*”. Additionally, reference lists from initially identified relevant articles were hand‐searched to identify further eligible studies. The full details of the search strategy are provided in Supplementary File Table S2.

### 2.2 Inclusion Criteria

(1) Population: Individuals diagnosed with T2DM, as reported in the original studies; (2) Intervention: BA or BA‐based interventions in which BA was the primary or core intervention component; (3) Control: For controlled studies, eligible comparators included treatment as usual, usual care, health education, guided exercise, or other non‐BA control conditions. Uncontrolled studies were summarized narratively and excluded from the meta‐analysis; (4) Outcomes: at least one of the following outcomes was reported: depressive symptoms, HbA1c, diabetes self‐efficacy, or diabetes self‐management behaviors; (5) Study Design: Interventional studies, including randomized controlled trials (RCTs) and non‐randomized interventional studies.

### 2.3 Exclusion Criteria

(1) Clinical expert opinions, clinical trial protocols, conference abstracts, animal studies, review articles, and non‐peer‐reviewed publications were excluded; (2) Studies not involving BA or BA‐based interventions as a primary or core intervention component; (3) Duplicate publications or studies with insufficient data; (4) Studies lacking full‐text availability; (5) Publications not written in English or Chinese.

### 2.4 Study Selection and Data Extraction

Following the importation of all retrieved records into EndNote21 and the removal of duplicates, two independent reviewers performed literature screening according to predefined inclusion and exclusion criteria. Any disagreements between the reviewers were resolved through discussion with a third researcher. The extracted data encompassed the following variables: author, publication year, country, study design, funding, participant characteristics (including sample size, age, gender, and inclusion criteria), as well as outcomes and intervention characteristics (such as delivery format, dosage, provider, core components, and any distinctive integration with diabetes care).

### 2.5 Risk‐of‐Bias and Certainty‐of‐Evidence Assessment

Two independent reviewers assessed the risk of bias of all included studies. For RCTs, the Cochrane Risk of Bias 2 tool (RoB 2) was used to assess bias arising from the randomization process, deviations from intended interventions, missing outcome data, outcome measurement, and selection of the reported result (Sterne et al. 2019). Each domain was rated as “low risk,” “some concerns,” or “high risk.” Non‐randomized interventional studies were evaluated using the ROBINS‐I tool (Sterne et al. 2016). Disagreements were resolved through discussion with a third reviewer.

The certainty of evidence for each meta‐analyzed outcome was assessed using the GRADE approach (Guyatt et al. 2011). Because the quantitative synthesis was based on RCT evidence, the initial certainty rating was high. Certainty was downgraded, where appropriate, according to five domains: risk of bias, inconsistency, indirectness, imprecision, and publication bias. The final certainty of evidence was rated as high, moderate, low, or very low.

### 2.6 Statistical Analysis

Quantitative meta‐analyses were restricted to RCTs to ensure methodological robustness. Meta‐analyses were performed using RevMan 5.4. For HbA1c, mean differences (MDs) with 95% confidence intervals (CIs) were calculated because all studies reported HbA1c in percentage units. For depressive symptoms, diabetes self‐efficacy, and diabetes self‐management behaviors, Hedges’ *g* was used as the standardized mean difference (SMD) to account for differences in measurement scales and small sample sizes. For the meta‐analyses, we used change‐from‐baseline data when available. When a report provided results from independent samples, these were treated as separate datasets; overlapping samples were not double‐counted. The data type, time point, outcome measure, and effect measure used for each analysis are reported in Supplementary Table S5. A random‐effects model was employed to account for expected clinical and methodological heterogeneity. Heterogeneity was assessed using Cochran's Q test, and an *I* ^2^ value of >50% was considered to indicate substantial heterogeneity. Sensitivity analyses using the leave‐one‐out method were conducted to examine the robustness of the pooled results. Exploratory subgroup analyses stratified by measurement scale were conducted for outcomes assessed using different instruments. Given the small number of datasets within each subgroup, these analyses were interpreted descriptively.

Publication bias was assessed using funnel plots and Egger's regression test for outcomes with at least five datasets. Egger's regression tests were performed using R software with the meta package. Egger's test was not performed for outcomes with fewer than five datasets. Given the small number of datasets included in each meta‐analysis, the results were interpreted cautiously.

### 2.7 Narrative Synthesis

To supplement the quantitative synthesis, a narrative synthesis was conducted for all included studies to describe the implementation characteristics of BA or BA‐based interventions in T2DM, including delivery format, intervention dosage, provider characteristics, core BA components, and integration with diabetes‐specific content.

## 3 Results

### 3.1 Literature Screening Process and Results

**FIGURE 1:**
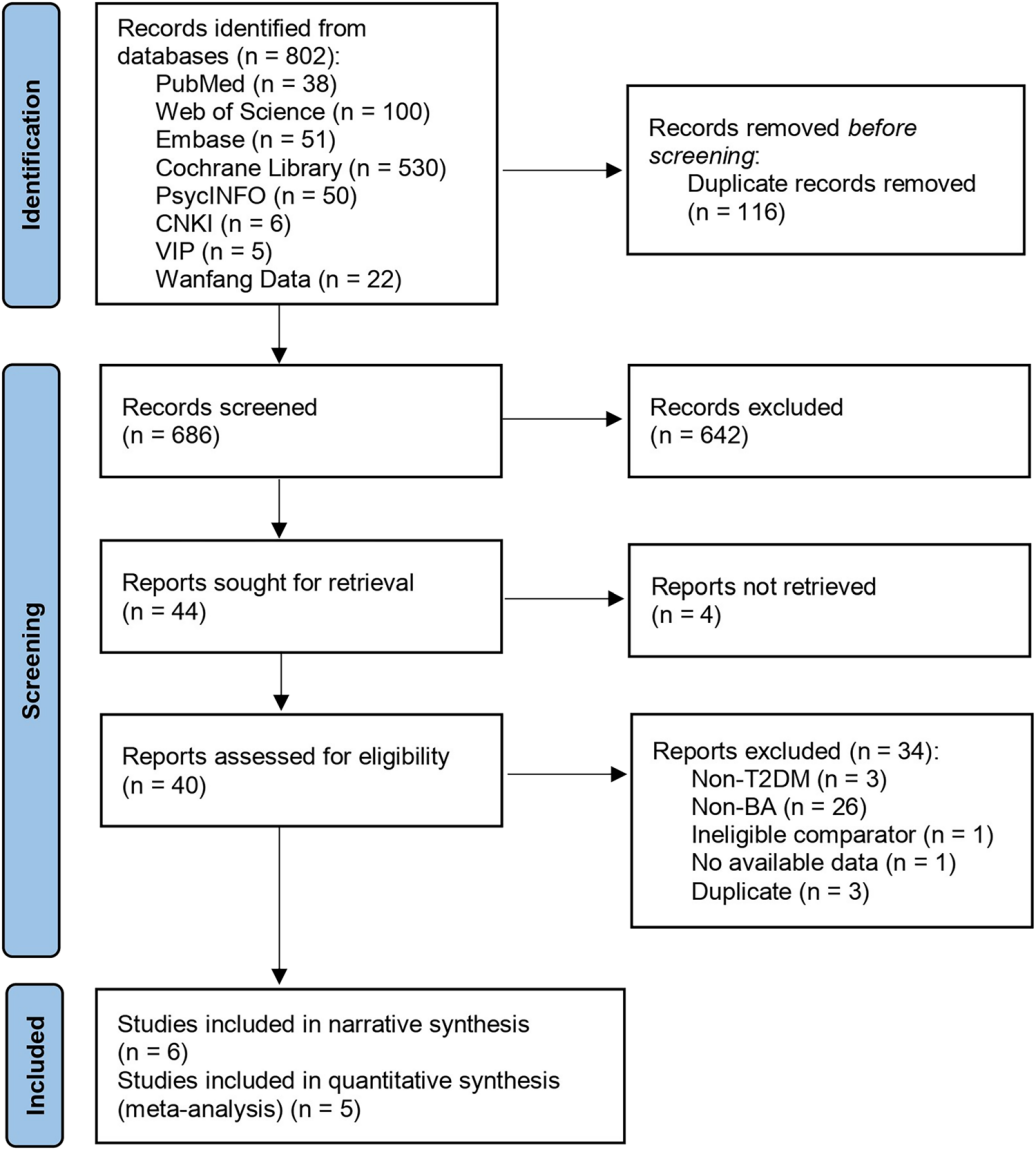
Literature screening process.

A total of 802 records were identified from database searches. After removing 116 duplicates, 686 records were screened by title and abstract, and 642 records were excluded. The remaining 44 reports were sought for retrieval, of which 4 were not retrieved. A full‐text eligibility assessment was conducted for 40 reports, and 34 reports were excluded with reasons listed in Supplementary File Table S3. Finally, 6 studies were included in the narrative synthesis, of which 5 RCTs were included in the quantitative meta‐analysis (Figure 1).

### 3.2 Characteristics of Included Studies

The characteristics of the included studies are summized in Table 1. A total of 6 studies published from 2015 to 2025 satisfied the inclusion criteria, comprising 5 RCTs and 1 pre‐post study. These studies collectively included a total of 252 persons diagnosed with T2DM, among whom 85.7% presented with depressive symptoms, and 55.2% met the criteria for major depressive disorder (MDD) as determined by the Structured Clinical Interview for DSM‐IV Disorders (SCID‐IV) or Mini‐International Neuropsychiatric Interview. BA was a fundamental element integrated into all intervention protocols.

**TABLE 1:** Characteristics of included studies.

Author/year	Country	Study type	Conditions	Additional eligibility criteria	Total sample, T/C	Age (mean ± SD)	Gender (% male)	Intervention, T	Intervention, C	Dropout (T/C)	Outcomes	Follow‐up timepoints
Li (2021)	China	RCT	T2DM	4 < PHQ‐9 < 10, HbA1c ≥ 7%	20, 11/9	T: 35.00 ± 7.2, C: 37.22 ± 6.10	65	BA‐based exercise intervention	TAU‐based Guided exercise	2/4	Depression, HbA1c	Baseline, post‐intervention (NR)
Ahmed et al. (2025)	Bangladesh	RCT	T2DM	PHQ‐9 > 4 and MDD	110, 51/59	49.6 ± 10.4	27.8	BA	TAU	9/2	Depression, HbA1c	Baseline, post‐intervention (12 weeks)
Bae et al. (2024)	Korea	RCT	T2DM	4 < PHQ‐9 < 20	39, 20/19	T: 60.4 ± 4.3, C: 63.9 ± 5.1	51.3	App‐based BA	TAU	19/15	Depression, HbA1c	Baseline, post‐intervention (12 weeks)
Kaltman et al. (2016)	USA	Pre‐post	T2DM	PHQ‐9 > 9, HbA1c ≥ 8%	18	49.7 ± 8.8	44	BA‐informed comprehensive behavioral intervention	N/A	5	Depression, HbA1c	Baseline, post‐intervention (6–14 weeks)
Schneider et al. (2016)	USA	RCT	T2DM	7 ≤ HbA1c ≤ 10%, MDD	29, 15/14	53.4 ± 7.1	0	BA‐based exercise intervention	TAU‐based Guided exercise	2/3	Depression, HbA1c	Baseline, post‐intervention (24 weeks)
Vickery et al. (2024)	USA	RCT	T2DM	HbA1c ≥ 7.5%, experience of homelessness in the past 24 months	36, 18/18	T: 52.2 ± 10.2; C: 51.9 ± 10.8	69.4	BA	TAU	0/2	HbA1c	Baseline, post‐intervention (12 weeks)

Abbreviations: BA = behavioral activation; C = control group; MDD = major depressive disorder; N/A = not applicable; NR = not reported. *Note*: PHQ‐9: Patient Health Questionnaire‐9, a self‐report measure of depression severity. Established clinical thresholds are: 0–4 (None), 5–9 (Mild), 10–14 (Moderate), 15–19 (Moderately severe), 20–27 (Severe); PHQ‐9 = Patient Health Questionnaire‐9; Pre‐post = pre‐test‐post‐test design; RCT = randomized controlled trial; T = treatment group; T2DM = type 2 diabetes mellitus; TAU = treatment as usual.

### 3.3 Risk of Bias of Included Studies

**FIGURE 2:**
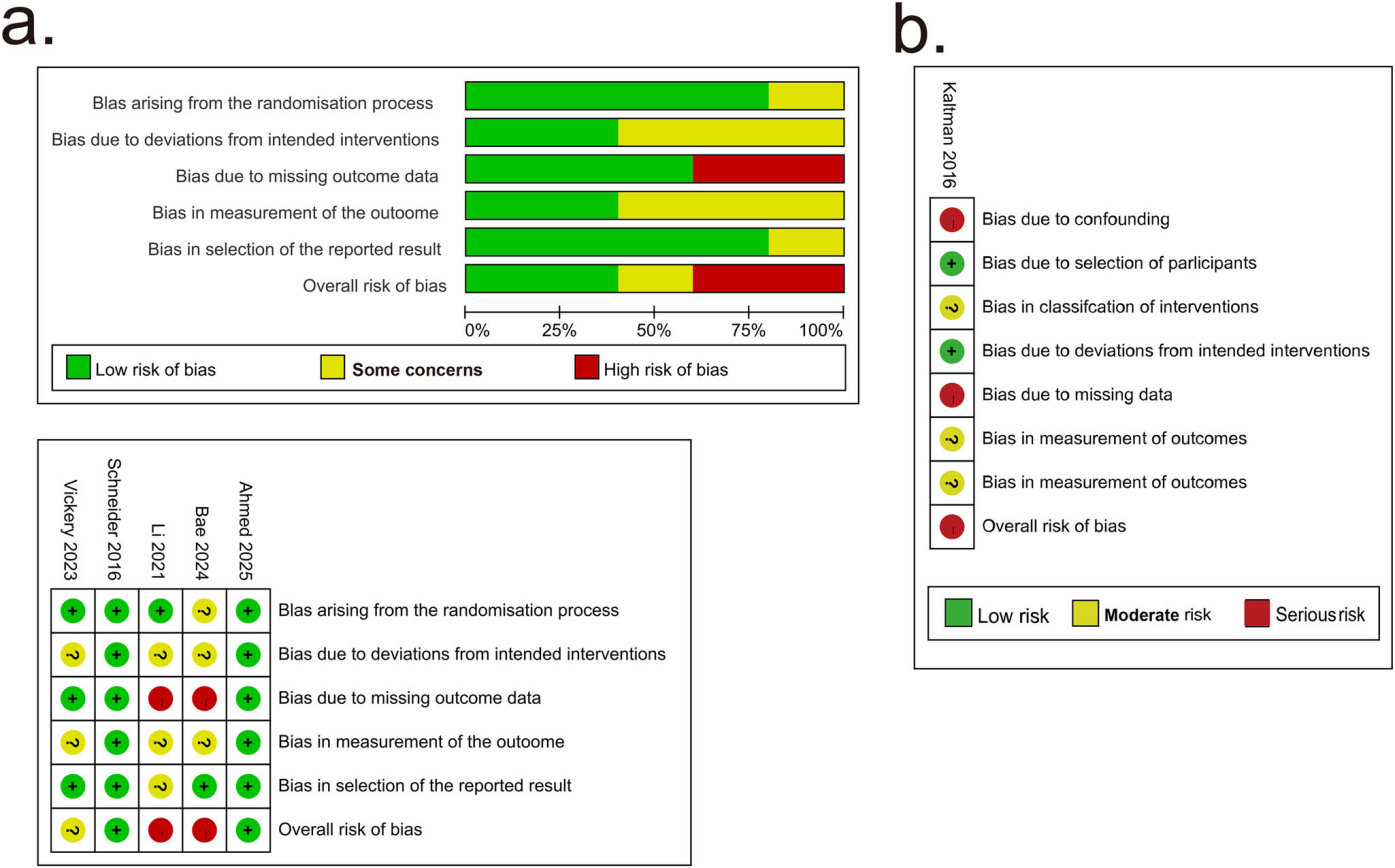
Risk‐of‐bias assessment of the included studies.

Of the 6 included studies, 5 RCTs were assessed using RoB 2, and 1 non‐randomized interventional study was evaluated using ROBINS‐I. Overall, 2 studies were rated as having low risk of bias, 1 study as having some concerns, and 3 studies as having high risk of bias. Among the 5 RCTs, 2 were rated as low risk, 1 as some concerns due to potential bias in outcome measurement, and 2 as high risk mainly because of missing outcome data. The non‐randomized study was rated as high risk due to concerns related to confounding and incomplete data (Figure 2).

### 3.4 Meta‐analysis of RCTs

#### 3.4.1 Depressive Symptoms

**FIGURE 3:**
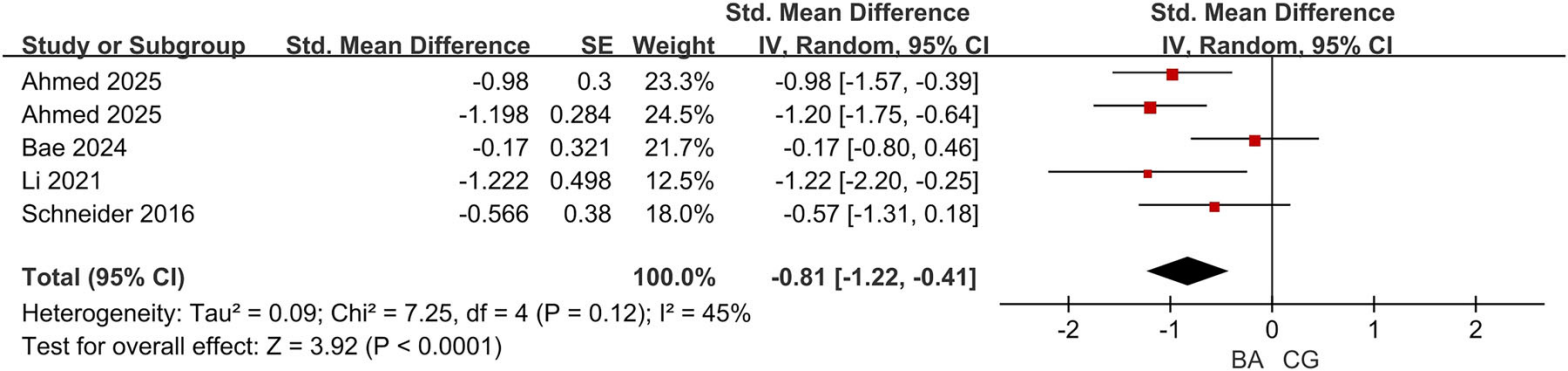
Forest plot of the effect of BA on depressive symptoms in people with T2DM.

4 studies provided depression outcome data, contributing 5 datasets to the meta‐analysis. The pooled analysis suggested a statistically significant reduction in depressive symptoms among patients with T2DM, with no substantial heterogeneity between studies (SMD = −0.81, 95% CI: −1.22 to −0.41, *p* < 0.001; *I* ^2^ = 45%; Figure 3).

Leave‐one‐out sensitivity analyses showed that the pooled effect on depressive symptoms remained statistically significant after omitting each study in turn (Supplementary Table S6).

#### 3.4.2 Glycemic Control (HbA1c)

**FIGURE 4:**
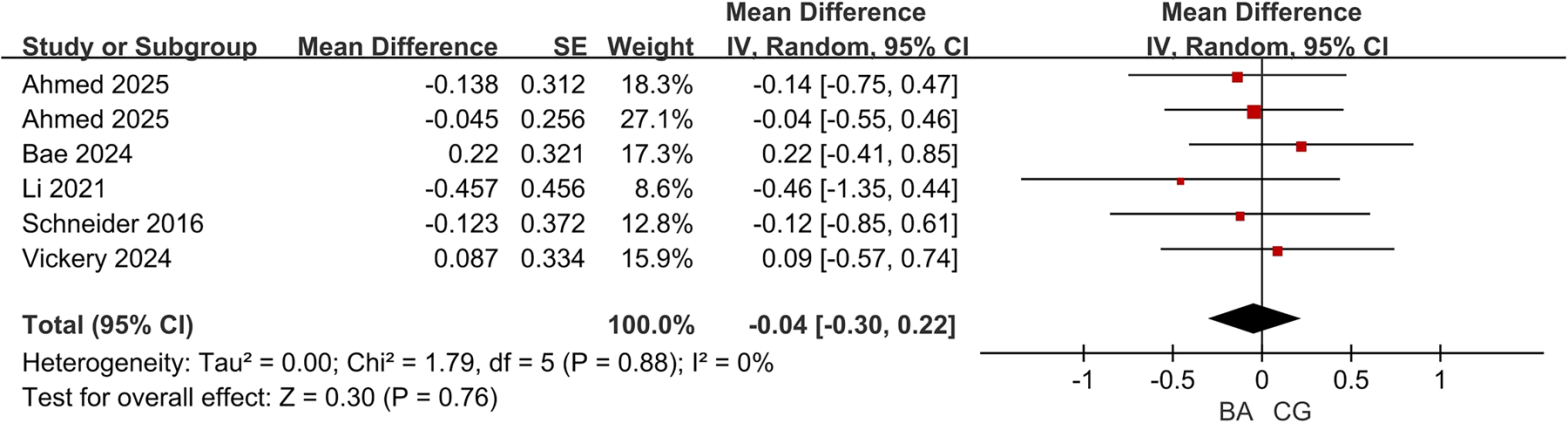
Forest plot of the effect of BA on HbA1c in people with T2DM.

5 studies provided HbA1c data, contributing 6 datasets to the meta‐analysis. The pooled analysis did not show a statistically significant effect of BA on HbA1c, with no significant heterogeneity observed between studies (MD = −0.04%, 95% CI: −0.30 to 0.22, *p* = 0.76; *I* ^2^ = 0%; Figure 4).

Leave‐one‐out sensitivity analyses showed that the pooled effect on HbA1c remained non‐significant after omitting each study in turn (Supplementary Table S6).

#### 3.4.3 Diabetes Self‐Efficacy and Self‐Management Behaviors

**FIGURE 5:**
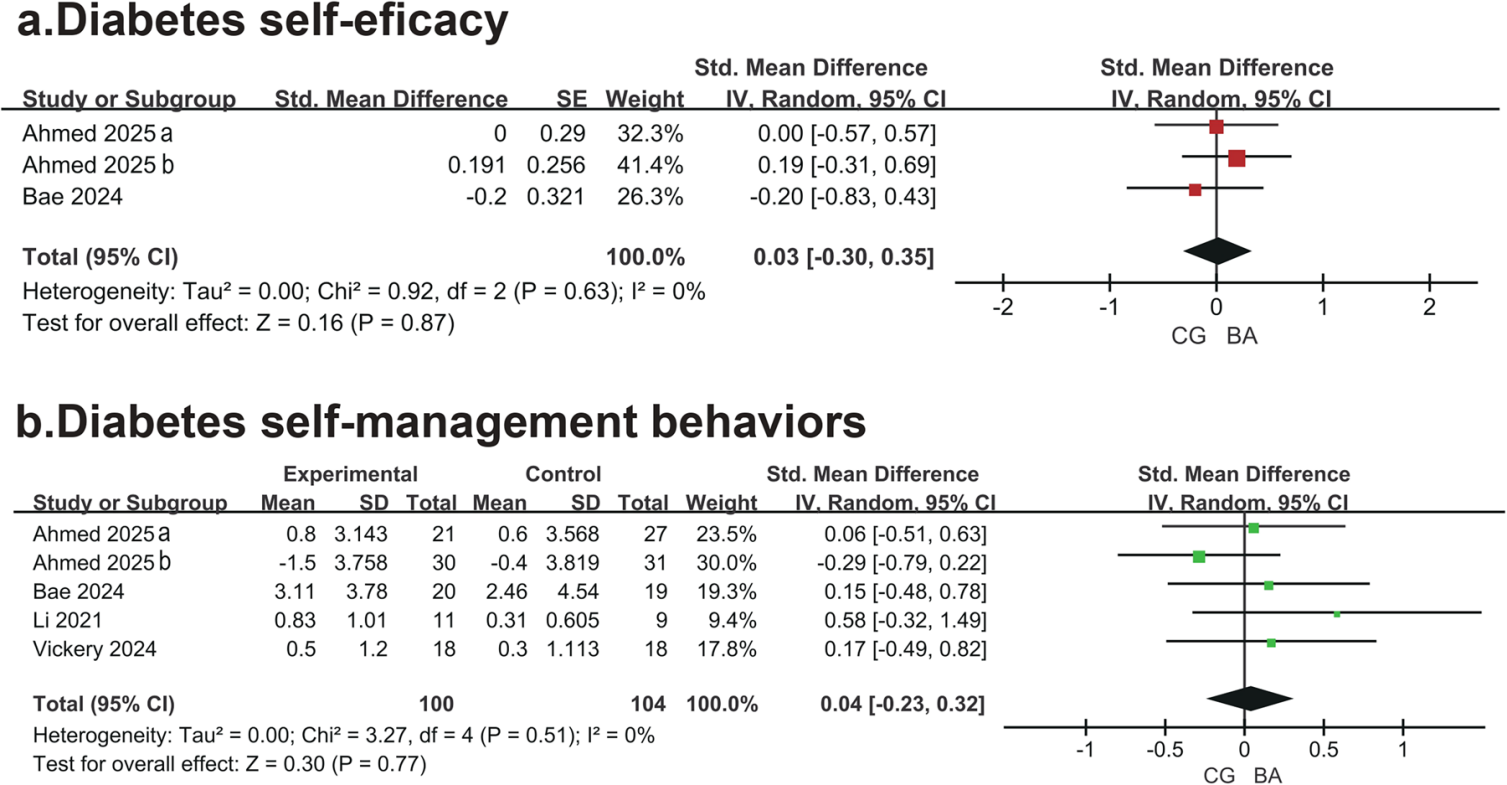
Forest plot of the effect of BA on diabetes self‐efficacy and self‐management behaviors.

Meta‐analyses of outcomes showed that the pooled effects were not statistically significant for diabetes self‐efficacy (SMD = 0.03, 95% CI: −0.30 to 0.35, *p* = 0.87; *I* ^2^ = 0%) or self‐management behaviors (SMD = 0.04, 95% CI: −0.23 to 0.32, *p* = 0.77; *I* ^2^ = 0%; Figure 5).

The non‐significant findings for diabetes self‐efficacy and self‐management behaviors were also unchanged in leave‐one‐out sensitivity analyses (Supplementary Table S6).

#### 3.4.4 Exploratory Subgroup Analyses by Measurement Scale

Exploratory subgroup analyses stratified by measurement scale were conducted for depressive symptoms, diabetes self‐efficacy, and diabetes self‐management behaviors. The corresponding subgroup forest plots are presented in Supplementary Figure S1–S3. For depressive symptoms, the PHQ‐9 subgroup showed a statistically significant reduction, whereas the HAMD subgroup did not; however, the test for subgroup differences did not indicate clear evidence of scale‐related differences (*p* = 0.51). For diabetes self‐efficacy and diabetes self‐management behaviors, the subgroup analyses by measurement scale also did not indicate clear evidence of subgroup differences (*p* = 0.41 and *p* = 0.48, respectively). Overall, these exploratory subgroup analyses did not materially change the interpretation of the main findings.

#### 3.4.5 Publication Bias and Certainty of Evidence

Funnel plots are presented in Supplementary Figure S4–S6. Egger's regression test did not indicate statistically significant funnel plot asymmetry for depressive symptoms, HbA1c, or diabetes self‐management behaviors (Supplementary Table S7). Egger's test was not performed for diabetes self‐efficacy because fewer than five datasets were available. Given the small number of datasets, these findings should be interpreted cautiously.

The GRADE assessment is presented in Supplementary Table S8. The certainty of evidence was rated as low for depressive symptoms, HbA1c, and diabetes self‐management behaviors, and very low for diabetes self‐efficacy. The certainty was mainly downgraded for risk of bias and imprecision. Risk‐of‐bias concerns reflected the presence of some studies with “some concerns” or “high risk” ratings, particularly due to missing outcome data or outcome measurement concerns. Imprecision was mainly related to the small number of studies and participants contributing to each pooled estimate; for non‐significant outcomes, the confidence intervals also crossed the line of no effect.

### 3.5 Findings From the Non‐Randomized Interventional Study

Kaltman et al. (2016) was retained for narrative synthesis because BA was described as a structured component of the intervention, but it was not included in the quantitative synthesis because it used a single‐arm pre‐post design. In the full sample (*N* = 18), HbA1c decreased from 9.6% to 8.8%, depressive symptoms decreased from 24.33 to 12.67, and diabetes‐related self‐efficacy increased from 6.83 to 8.72; improvements were also reported in diet, glucose monitoring, and foot care. These findings should be interpreted cautiously because of the uncontrolled design and small sample size.

### 3.6 Narrative Synthesis of BA Intervention Characteristics

The key characteristics of the BA interventions are summarized in Table 2, with emphasis on delivery parameters, provider characteristics, core components, and integration with diabetes care. This synthesis highlights both the consistent application of BA core principles and the diversity in its implementation within diabetes management contexts.

**TABLE 2:** Characteristics of behavioral activation interventions in the included studies.

Author/year	Delivery format	Duration, week	Total sessions	Length per session, min	Provider characteristics	Activity monitoring	Value assessment/goal setting	Activation strategies	Maintenance strategies	Relapse prevention	Combined intervention	Diabetes‐specific
Li (2021)	Group face‐to‐face	NR	8	90	Nurse, sports rehabilitation therapists	Yes	Yes	a), c)	b)	NR	Exercise program	Pre‐exercise sports medicine examination and safety assessment; guidance on safe exercise for diabetes
Ahmed et al. (2025)	Individual face‐to‐face or remotely	6–12	6	20–40	Non‐mental health specialist	Yes	Yes	NR	a), b)	NR	No	Integrate the content of self‐management for diabetes and blood glucose monitoring
Bae et al. (2024)	Mobile app (Dangdang Care)	12	NR	NR	N/A	Yes	Yes	NR	b)	Yes	No	Setting and working towards goals deemed beneficial for managing diabetes
Kaltman et al. (2016)	Individual face‐to‐face	6–14	8	45	Chronic care managers from the primary care clinic	Yes	Yes	NR	NR	NR	Motivational interviewing	T2DM and depression management topics: activities, exercise, glucose monitoring, medication adherence, diet, stress management, social support
Schneider et al. (2016)	Group face‐to‐face	24	38	90	exercise professionals	Yes	Yes	NR	b)	NR	Exercise program	Pre‐exercise sports medicine examination and safety assessment; guidance on safe exercise for diabetes
Vickery et al. (2024)	Individual face‐to‐face and telephone meetings	12	10	30	Non‐mental health specialist	Yes	Yes	b)	b)	Yes	Motivational interviewing	Diabetes education, goal setting focused on blood sugar control goals (mostly diabetes pill medication adherence) and psychological wellness

Abbreviations: BA = behavioral activation; Maintenance strategies: a) addressing avoidance, b) barrier problem‐solving; N/A = not applicable. “Yes” indicates that the component was present or described in the study. “No” indicates that the component was explicitly absent or not mentioned. Notes: Activation strategies: a) activity grading, b) behavioral reward, c) behavioral contract; NR = not reported.

#### 3.6.1 Delivery Format and Dosage

The interventions were administered through diverse delivery formats, including individual face‐to‐face sessions, group‐based sessions, blended in‐person and remote delivery, telephone‐supported delivery, and mobile app‐based delivery.

Regarding dosage, the duration of interventions spanned from 6 to 24 weeks, with the total number of sessions ranging broadly between 6 and 38. The length of each session also exhibited considerable variation, from 20 to 90 min.

#### 3.6.2 Provider Characteristics and BA Core Components

A key finding was that the majority of interventions were delivered by non‐mental health specialists, including nurses, diabetes educators, exercise professionals, and chronic care managers.

All studies incorporated activity monitoring and goal setting, but activation strategies and maintenance strategies varied. Notably, only the studies by Bae et al. (2024) and Vickery et al. (2024) explicitly addressed relapse prevention.

#### 3.6.3 Integration With Combined Interventions and Diabetes‐Specific Content

The included studies varied in how BA was combined with other components. Some interventions paired BA with structured exercise programs or motivational interviewing, whereas others embedded BA techniques within diabetes‐specific self‐management content, such as medication adherence, dietary choices, and blood glucose monitoring. These features were summarized descriptively because the small number of studies did not support formal subgroup analyses comparing combined and non‐combined approaches.

## 4 Discussion

### 4.1 Effectiveness of BA on Depressive Symptoms in T2DM

The meta‐analysis suggested that BA may reduce depressive symptoms in individuals with T2DM. This outcome aligns with findings reported in other chronic disease populations, such as cancer and stroke (Yisma et al. 2024; Wei et al. 2025). BA functions by promoting engagement in rewarding activities and decreasing avoidance behaviors, thereby interrupting the vicious cycle of persistent depression (Hopko et al. 2003). In the context of T2DM, this finding suggests that BA may address a psychological barrier relevant to self‐management (Alexandre et al. 2021). Importantly, the included studies suggest that BA may reduce depressive symptoms even when diabetes management content is incorporated. In the study conducted by Kaltman et al. (2016), participants reported not only reduced depressive symptoms but also gained a deeper understanding of diabetes and felt more motivated for self‐management. This suggests that integrating diabetes‐related content into BA may be feasible without weakening its depression‐focused function, although more direct comparative evidence is needed.

### 4.2 Effectiveness of BA on HbA1c in T2DM

Theoretically, reductions in depressive symptoms through BA may improve diabetes‐related self‐efficacy, facilitate self‐management behaviors, and ultimately contribute to better glycemic control (Davis et al. 2022; Schmitt et al. 2021; Wojujutari Ajele and Sunday Idemudia 2025; Ting et al. 2025). However, the current meta‐analysis did not demonstrate statistically significant improvements in HbA1c, diabetes self‐efficacy, or diabetes self‐management behaviors. Given the small number of included studies and the low or very low certainty of evidence, these non‐significant findings should not be interpreted as definitive evidence that BA has no effect on these outcomes. Rather, they suggest that the available evidence remains insufficient to determine whether BA can reliably improve diabetes‐related behavioral and physiological outcomes in T2DM.

One possible explanation is that reductions in depressive symptoms alone may not be sufficient to produce measurable improvements in diabetes‐related confidence, self‐management behaviors, or glycemic control within the available intervention periods. This may be because diabetes self‐management behaviors are often complex and demanding (Carpenter et al. 2019). Unlike the inherently rewarding activities prioritized in standard BA, diabetes self‐management tasks may be experienced as burdensome, unrewarding, or difficult to sustain, thereby remaining targets of behavioral avoidance despite improvements in general mood (Iturralde et al. 2017). This challenge is further amplified in our included population, where 85.7% exhibited depressive symptoms and 55.2% were diagnosed with major depressive disorder (MDD), making sustained behavioral engagement more difficult for these individuals (Genis‐Mendoza et al. 2022).

Given that the current evidence did not demonstrate statistically significant effects on self‐efficacy or self‐management behaviors, which are potential pathways for glycemic outcomes, BA may need to be more explicitly directed toward activating and sustaining diabetes self‐management behaviors rather than focusing only on general engagement in positive activities. The limited available evidence suggests that this diabetes‐specific focus may be important, although findings remain inconsistent. For instance, Li (2021) targeted diabetes exercise goals (150 min/week of moderate‐intensity exercise) within the BA framework and reported significant reductions in HbA1c. Similarly, Vickery et al. (2024) focused on medication adherence, a behavior closely related to glycemic outcomes, and reported favorable glycemic changes. In contrast, Schneider et al. (2016) also focused on exercise goals but did not report significant improvements in HbA1c. One possible explanation is that the study was designed primarily as a feasibility trial, rather than to test whether exercise intensity was sufficient to produce physiological change. Together, these findings suggest that diabetes‐specific activation targets may be relevant, but target selection should be considered alongside intervention intensity, fidelity, feasibility, and follow‐up duration. This interpretation is consistent with broader findings from psychological intervention studies, in which interventions explicitly targeting diabetes management tend to produce greater glycemic benefits (Winkley et al. 2020; Wang et al. 2024; Ngan et al. 2021). Future interventions should not assume that mood improvement alone is sufficient for glycemic control; rather, BA principles should be strategically applied to activate specific, physiologically relevant self‐management behaviors.

### 4.3 Implementation Characteristics of BA Interventions

The narrative synthesis highlights the flexible delivery of BA and its potential for integration into diverse diabetes care settings. In this context, BA may be better understood as a behavioral support strategy rather than a replacement for traditional lifestyle interventions. Compared with education‐ or prescription‐based lifestyle programs, BA provides structured strategies such as activity monitoring, graded goal setting, problem‐solving, and reinforcement to help patients initiate and sustain self‐management behaviors (Fredrix et al. 2018; Tarricone et al. 2024).

First, BA appears adaptable in delivery format and intervention dosage, although adherence remains an important consideration. In T2DM populations, BA has been delivered individually or in groups, face‐to‐face or remotely, and either alone or in combination with diabetes‐related components. This flexibility may support adaptation to different clinical settings and patient preferences, but it also creates implementation challenges. For instance, remote or app‐based delivery may improve convenience, but the app‐based study included in this review also reported high dropout rates (Bae et al. 2024). BA interventions also varied in dosage, including intervention duration, total number of sessions, and session length. Previous BA research has not clearly established a simple dose‐response pattern in which more sessions are necessarily more effective than fewer sessions (Wei et al. 2025; Hershenberg et al. 2015). Shorter or lower‐burden formats may improve adherence for some patients by reducing intervention burden. This consideration is particularly relevant for chronic disease management, where sustained behavioral change is required. Future research should therefore compare delivery models and dosages not only in terms of efficacy, but also adherence, intervention burden, and cost‐effectiveness.

Second, BA may be scalable because it can be delivered by trained non‐mental health professionals. In the included studies, BA or BA‐based interventions were delivered by nurses, diabetes educators, exercise professionals, chronic care managers, or other trained non‐specialists. Although provider background may influence intervention delivery and outcomes (Winkley et al. 2020; Zu et al. 2024), broader evidence from task‐shifting and low‐intensity psychological intervention research suggests that appropriately trained non‐specialists can deliver structured psychological interventions (Alam et al. 2009; Ekers, Godfrey et al. 2011; Oyedeji et al. 2022). Compared with more complex psychological interventions, BA's relative simplicity may facilitate training and delivery by non‐specialists (Kanter et al. 2012). This feature is particularly relevant for resource‐constrained settings such as community health centers. By involving frontline providers who already maintain long‐term relationships with patients, BA may reduce reliance on separate specialist mental health visits and improve accessibility (Katon et al. 2008; Ekers, Richards et al. 2011).

Third, although core components such as activity monitoring and goal setting were consistently reported, specific activation and maintenance strategies varied widely and were often underreported. All included studies incorporated fundamental BA components, such as activity monitoring and goal setting (Kanter et al. 2010). Nevertheless, considerable variations were observed in specific activation strategies (e.g., activity grading, behavioral rewards, and behavioral contracts) and maintenance strategies (e.g., addressing avoidance and problem‐solving). Only a few studies explicitly mentioned relapse prevention strategies (Vickery et al. 2024; Bae et al. 2024), suggesting potential shortcomings in most BA interventions for maintaining long‐term effects. Furthermore, although most interventions included diabetes‐related content, the extent to which BA techniques were explicitly used to activate specific diabetes self‐management behaviors was often insufficiently described. Clearer reporting of these details is needed to determine which components are necessary for improving patient outcomes.

### 4.4 Limitations and Strengths

This study acknowledges several limitations that warrant consideration. First and foremost is the limited statistical power. The small number of included RCTs and the relatively small total sample size increase the risk of Type II errors, particularly for outcomes with high inter‐individual variability such as HbA1c. Second, the lack of long‐term follow‐up data represents a critical gap. Most included studies assessed outcomes immediately post‐intervention. Given that HbA1c reflects average blood glucose over the preceding three months (American Diabetes Association Professional Practice Committee 2025), short‐term interventions may not fully capture the physiological lag of behavioral changes. Furthermore, without extended follow‐up, we cannot determine whether the behavioral activation effects are sustainable or if patients relapse once the structured support ends. Third, clinical heterogeneity complicates the isolation of BA effects. Variations in delivery format, provider background, intervention dosage, combined components, behavioral targets, and implementation fidelity made it difficult to determine which features were most strongly associated with outcomes. The small number of studies also limited formal subgroup or moderator analyses. Finally, the risk of bias varied, with several studies rated as having “some concerns” or “high risk,” mainly due to missing outcome data and outcome measurement concerns.

Despite these shortcomings, the findings of this study provide useful insights for future research and intervention development. To our knowledge, this represents the first systematic review to quantitatively synthesize evidence specifically on the effectiveness of BA for individuals with T2DM. A key strength lies in the integration of meta‐analysis with a comprehensive narrative synthesis of intervention characteristics, thereby providing valuable insights into how BA was implemented and integrated into diabetes care contexts. This approach enabled a move beyond the question of “does it work” to preliminarily explore “how it might work” and “how it can be optimized,” particularly highlighting the role of non‐specialist delivery and the importance of integrating diabetes‐specific content. Furthermore, the low to moderate statistical heterogeneity supports the consistency of the pooled estimates, although the certainty of evidence remains limited by the small number of studies and risk‐of‐bias concerns.

## 5 Conclusion

This review suggests that BA may reduce depressive symptoms in individuals with T2DM, whereas its effects on glycemic control, diabetes self‐efficacy, and self‐management behaviors remain uncertain. The narrative synthesis indicates that BA has been delivered in flexible formats and may have potential for integration into routine diabetes care, particularly when delivered by trained non‐mental health professionals and combined with diabetes‐specific content. Future adequately powered RCTs with longer follow‐up should test whether embedding diabetes self‐management goals directly within the BA framework can improve behavioral and physiological outcomes.

## Author Contributions

**Xiao Lu**: conceptualization, investigation, methodology, writing – original draft, writing – review and editing, software, data curation, validation. **Zhuo Wang**: investigation, methodology, validation, formal analysis, writing – review and editing. **Tian Xia**: conceptualization, methodology, validation, data curation, writing – review and editing, formal analysis, investigation. **Rong Xu**: writing – review and editing, project administration, supervision, funding acquisition, conceptualization, methodology, formal analysis, resources. **Mengjiao Cheng**: validation, visualization, software, writing – original draft.

## Funding

This work was funded by Hubei Shizhen Laboratory (Grant No. SZL‐2025‐KF‐202), and the Nursing Special Fund (Major Project) of Tongji Hospital, Tongji Medical College of Huazhong University of Science and Technology (Grant No. 2023C08).

## Ethics Statement

The study did not require ethical approval and complied with the journal's ethics policy as it was based on analysis of existing literature and did not involve new data collection or human participants.

## Conflicts of Interest

The authors declare no conflicts of interest.

## Supporting information




**Supplementary Materials**: brb371761‐sup‐0001‐SuppMat.docx

## Data Availability

The data that support the findings of this study are openly available in the included studies.
