# Supplementary material for: Behavioral Activation for Depression and Glycemic Control in Type 2 Diabetes: A Systematic Review and Meta‐Analysis
Source: Brain Behav. 2026 Sep 15;16(9):e71761. doi: 10.1002/brb3.71761 (PMC13575635; doi:10.1002/brb3.71761)
Supplement: Supplementary file 1 [file BRB3-16-e71761-s001.docx]

**Table S1.** **Prisma checklist**

| **Section and Topic** | **Item #** | **Checklist item** | **Location where item is reported** |
| --- | --- | --- | --- |
| **TITLE** | | |  |
| Title | 1 | Identify the report as a systematic review. | P.1 |
| **ABSTRACT** | | |  |
| Abstract | 2 | See the PRISMA 2020 for Abstracts checklist. | P.1-3 |
| **INTRODUCTION** | | |  |
| Rationale | 3 | Describe the rationale for the review in the context of existing knowledge. | P.3-4 |
| Objectives | 4 | Provide an explicit statement of the objective(s) or question(s) the review addresses. | P.4 |
| **METHODS** | | |  |
| Eligibility criteria | 5 | Specify the inclusion and exclusion criteria for the review and how studies were grouped for the syntheses. | P.5-6 |
| Information sources | 6 | Specify all databases, registers, websites, organisations, reference lists and other sources searched or consulted to identify studies. Specify the date when each source was last searched or consulted. | P.5 |
| Search strategy | 7 | Present the full search strategies for all databases, registers and websites, including any filters and limits used. | P 5, Supplementary File Table S2 |
| Selection process | 8 | Specify the methods used to decide whether a study met the inclusion criteria of the review, including how many reviewers screened each record and each report retrieved, whether they worked independently, and if applicable, details of automation tools used in the process. | P.6 |
| Data collection process | 9 | Specify the methods used to collect data from reports, including how many reviewers collected data from each report, whether they worked independently, any processes for obtaining or confirming data from study investigators, and if applicable, details of automation tools used in the process. | P.6 |
| Data items | 10a | List and define all outcomes for which data were sought. Specify whether all results that were compatible with each outcome domain in each study were sought (e.g. for all measures, time points, analyses), and if not, the methods used to decide which results to collect. | P.6 |
|  | 10b | List and define all other variables for which data were sought (e.g. participant and intervention characteristics, funding sources). Describe any assumptions made about any missing or unclear information. | P.6 |
| Study risk of bias assessment | 11 | Specify the methods used to assess risk of bias in the included studies, including details of the tool(s) used, how many reviewers assessed each study and whether they worked independently, and if applicable, details of automation tools used in the process. | P.6-7 |
| Effect measures | 12 | Specify for each outcome the effect measure(s) (e.g. risk ratio, mean difference) used in the synthesis or presentation of results. | P.7 |
| Synthesis methods | 13a | Describe the processes used to decide which studies were eligible for each synthesis (e.g. tabulating the study intervention characteristics and comparing against the planned groups for each synthesis (item #5)). | P.7-8 |
|  | 13b | Describe any methods required to prepare the data for presentation or synthesis, such as handling of missing summary statistics, or data conversions. | P.7-8 |
|  | 13c | Describe any methods used to tabulate or visually display results of individual studies and syntheses. | P.7-8 |
|  | 13d | Describe any methods used to synthesize results and provide a rationale for the choice(s). If meta-analysis was performed, describe the model(s), method(s) to identify the presence and extent of statistical heterogeneity, and software package(s) used. | P.7-8 |
|  | 13e | Describe any methods used to explore possible causes of heterogeneity among study results (e.g. subgroup analysis, meta-regression). | P.7-8, Supplementary Figures S1–S3 |
|  | 13f | Describe any sensitivity analyses conducted to assess robustness of the synthesized results. | P.8 |
| Reporting bias assessment | 14 | Describe any methods used to assess risk of bias due to missing results in a synthesis (arising from reporting biases). | P.8 |
| Certainty assessment | 15 | Describe any methods used to assess certainty (or confidence) in the body of evidence for an outcome. | P.8 |
| **RESULTS** | | |  |
| Study selection | 16a | Describe the results of the search and selection process, from the number of records identified in the search to the number of studies included in the review, ideally using a flow diagram. | P.8-9, Figure 1 |
|  | 16b | Cite studies that might appear to meet the inclusion criteria, but which were excluded, and explain why they were excluded. | Supplementary File Table S3 |
| Study characteristics | 17 | Cite each included study and present its characteristics. | P9-10, Table 1 |
| Risk of bias in studies | 18 | Present assessments of risk of bias for each included study. | P.11, Figure 2 |
| Results of individual studies | 19 | For all outcomes, present, for each study: (a) summary statistics for each group (where appropriate) and (b) an effect estimate and its precision (e.g. confidence/credible interval), ideally using structured tables or plots. | P.11-13, Figure 3-5 |
| Results of syntheses | 20a | For each synthesis, briefly summarise the characteristics and risk of bias among contributing studies. | P.13-14 |
|  | 20b | Present results of all statistical syntheses conducted. If meta-analysis was done, present for each the summary estimate and its precision (e.g. confidence/credible interval) and measures of statistical heterogeneity. If comparing groups, describe the direction of the effect. | P.11-13, Figure 3-5 |
|  | 20c | Present results of all investigations of possible causes of heterogeneity among study results. | P.11-13 |
|  | 20d | Present results of all sensitivity analyses conducted to assess the robustness of the synthesized results. | P.11-13 |
| Reporting biases | 21 | Present assessments of risk of bias due to missing results (arising from reporting biases) for each synthesis assessed. | P.13-14, Supplementary Figures S4–S6 |
| Certainty of evidence | 22 | Present assessments of certainty (or confidence) in the body of evidence for each outcome assessed. | P.13-14 |
| **DISCUSSION** | | |  |
| Discussion | 23a | Provide a general interpretation of the results in the context of other evidence. | P.18-22 |
|  | 23b | Discuss any limitations of the evidence included in the review. | P.22-23 |
|  | 23c | Discuss any limitations of the review processes used. | P.22-23 |
|  | 23d | Discuss implications of the results for practice, policy, and future research. | P.18-23 |
| **OTHER INFORMATION** | | |  |
| Registration and protocol | 24a | Provide registration information for the review, including register name and registration number, or state that the review was not registered. | P 4 |
|  | 24b | Indicate where the review protocol can be accessed, or state that a protocol was not prepared. | P.4 |
|  | 24c | Describe and explain any amendments to information provided at registration or in the protocol. | P.4 |
| Support | 25 | Describe sources of financial or non-financial support for the review, and the role of the funders or sponsors in the review. | P 24 |
| Competing interests | 26 | Declare any competing interests of review authors. | P.24 |
| Availability of data, code and other materials | 27 | Report which of the following are publicly available and where they can be found: template data collection forms; data extracted from included studies; data used for all analyses; analytic code; any other materials used in the review. | P.24 |

*From:*  Page MJ, McKenzie JE, Bossuyt PM, Boutron I, Hoffmann TC, Mulrow CD, et al. The PRISMA 2020 statement: an updated guideline for reporting systematic reviews. BMJ 2021;372:n71. doi: 10.1136/bmj.n71

**Table S2. The detailed search strategy**

| **Database** | **Search Strategy** |
| --- | --- |
| Pubmed | #1="Diabetes Mellitus, Type 2"[MeSH Terms] OR "Diabetes Mellitus, Type 2"[Title/Abstract] OR "T2DM"[Title/Abstract] OR "Noninsulin Dependent Diabetes Mellitus"[Title/Abstract] OR "Non-Insulin-Dependent Diabetes Mellitus"[Title/Abstract] OR "Maturity-Onset Diabetes Mellitus"[Title/Abstract] OR "Adult-Onset Diabetes Mellitus"[Title/Abstract] OR "Ketosis-Resistant Diabetes Mellitus"[Title/Abstract] OR "NIDDM"[Title/Abstract] OR "Type 2 Diabetes"[Title/Abstract] OR ("type 2"[Title/Abstract] AND ("diabet*"[Title/Abstract] OR "Diabetes Mellitus"[Title/Abstract]))  #2=("behavior* activat*"[Title/Abstract] OR "behaviour* activat*"[Title/Abstract] OR "behavioral activation"[Title/Abstract] OR "behavioural activation"[Title/Abstract]) OR "activit* schedul*"[Title/Abstract] OR (("pleasant"[Title/Abstract] OR "pleasurable"[Title/Abstract] OR "rewarding"[Title/Abstract]) AND ("activit*"[Title/Abstract] OR "event*"[Title/Abstract])) OR ("BA program*"[Title/Abstract] OR "BA protocol*"[Title/Abstract])  #3=#1 AND #2 |
| Embase | #1='non insulin dependent diabetes mellitus'/exp OR 'type 2 diabetes mellitus'/exp OR 'maturity onset diabetes'/exp OR 'adult onset diabetes mellitus'/exp  #2="type 2 diabetes":ti,ab OR "T2DM":ti,ab OR "NIDDM":ti,ab OR "non insulin dependent diabetes":ti,ab OR ("type 2" NEAR/3 diabet*):ti,ab  #3=#1 OR #2  #4='behavior* activat*':ti,ab OR 'behaviour* activat*':ti,ab OR 'behavioral activation':ti,ab OR 'behavioural activation':ti,ab OR 'activit* schedul*':ti,ab OR ('pleasant':ti,ab OR 'pleasurable':ti,ab OR 'rewarding':ti,ab) AND ('activit*':ti,ab OR 'event*':ti,ab) OR 'BA program*':ti,ab OR 'BA protocol*':ti,ab  #5=#3 AND #4 |
| Web of Science | #1=TS=("Diabetes Mellitus, Type 2" OR "T2DM" OR "Noninsulin Dependent Diabetes Mellitus" OR "Non-Insulin-Dependent Diabetes Mellitus" OR "Maturity-Onset Diabetes Mellitus" OR "Adult-Onset Diabetes Mellitus" OR "Ketosis-Resistant Diabetes Mellitus" OR "NIDDM" OR "Type 2 Diabetes" OR ("Type 2" NEAR/3 (Diabet* OR "Diabetes Mellitus")))  #2=TS=("behavior* activat*" OR "behaviour* activat*" OR "behavioral activation" OR "behavioural activation" OR "activit* schedul*" OR ("pleasant" OR "pleasurable" OR "rewarding") AND ("activit*" OR "event*") OR "BA program*" OR "BA protocol*")  #3=#1 AND #2 |
| Cochrane Library | #1=[MeSH DESCRIPTOR "Diabetes Mellitus, Type 2" EXPLODE ALL]  #2="diabetes mellitus, type 2":ti,ab,kw OR "t2dm":ti,ab,kw OR "noninsulin dependent diabetes mellitus":ti,ab,kw OR "non-insulin-dependent diabetes mellitus":ti,ab,kw OR "maturity-onset diabetes mellitus":ti,ab,kw OR "adult-onset diabetes mellitus":ti,ab,kw OR "ketosis-resistant diabetes mellitus":ti,ab,kw OR "niddm":ti,ab,kw OR "type 2 diabetes":ti,ab,kw OR ("type 2" NEAR/3 diabet*):ti,ab,kw  #3=#1 OR #2  #4= MeSH descriptor: Behavioral Activation explode all trees  #5=(behavior* activat*):ti,ab,kw OR (behaviour* activat*):ti,ab,kw OR (behavioral activation):ti,ab,kw OR (behavioural activation):ti,ab,kw OR (BA program*:ti,ab,kw) OR (BA protocol*:ti,ab,kw)  #6=#4 OR #5  #7=(activit* schedul*):ti,ab,kw  #8=(pleasant:ti,ab,kw OR pleasurable:ti,ab,kw OR rewarding:ti,ab,kw) AND (activit*:ti,ab,kw OR event*:ti,ab,kw)  #9=#7 OR #8  #10=#6 OR #9  #11=#3 AND #10 |
| PsycINFO | #1= MM "Diabetes Mellitus, Type 2" OR TI "Diabetes Mellitus, Type 2" OR AB "Diabetes Mellitus, Type 2" OR TI T2DM OR AB T2DM OR TI "Noninsulin Dependent Diabetes Mellitus" OR AB "Noninsulin Dependent Diabetes Mellitus" OR TI "Non-Insulin-Dependent Diabetes Mellitus" OR AB "Non-Insulin-Dependent Diabetes Mellitus" OR TI "Maturity-Onset Diabetes Mellitus" OR AB "Maturity-Onset Diabetes Mellitus" OR TI "Adult-Onset Diabetes Mellitus" OR AB "Adult-Onset Diabetes Mellitus" OR TI "Ketosis-Resistant Diabetes Mellitus" OR AB "Ketosis-Resistant Diabetes Mellitus" OR TI NIDDM OR AB NIDDM OR TI "Type 2 Diabetes" OR AB "Type 2 Diabetes" OR (TI ("type 2" AND ("diabet*" OR "Diabetes Mellitus")) OR AB ("type 2" AND ("diabet*" OR "Diabetes Mellitus")))  #2=(TI(behavior* activat*) OR AB(behavior* activat*) OR TI(behaviour* activat*) OR AB(behaviour* activat*) OR TI(behavioral activation) OR AB(behavioral activation) OR TI(behavioural activation) OR AB(behavioural activation)) OR (TI(activit* schedul*) OR AB(activit* schedul*)) OR ((TI(pleasant) OR AB(pleasant) OR TI(pleasurable) OR AB(pleasurable) OR TI(rewarding) OR AB(rewarding)) AND (TI(activit*) OR AB(activit*) OR TI(event*) OR AB(event*))) OR (TI("BA program*") OR AB("BA program*") OR TI("BA protocol*") OR AB("BA protocol*"))#3=#1 AND #2 |
| CNKI | (篇关摘：（2型糖尿病）OR 篇关摘：（T2DM）OR 篇关摘：（非胰岛素依赖型糖尿病）OR 篇关摘：（成人发病型糖尿病）OR 篇关摘：（NIDDM）) AND (篇关摘：（行为激活）OR 篇关摘：（行为活化） OR 篇关摘：（活动监测） OR 篇关摘：（目标设定）OR 篇关摘：（活动安排）OR 篇关摘：（BA疗法） |
| VIP | (题目或关键词：（2型糖尿病）OR 题目或关键词：（T2DM）OR 题目或关键词：（非胰岛素依赖型糖尿病）OR 题目或关键词：（成人发病型糖尿病）) AND (题目或关键词：（行为激活）OR 题目或关键词：（行为活化） OR 题目或关键词：（活动监测） OR 题目或关键词：（目标设定）OR 题目或关键词：（活动安排）OR 题目或关键词：（BA疗法） |
| WanFang Date | (题目或关键词：（2型糖尿病）OR 题目或关键词：（T2DM）OR 题目或关键词：（非胰岛素依赖型糖尿病）OR 题目或关键词：（成人发病型糖尿病）) AND (题目或关键词：（行为激活）OR 题目或关键词：（行为活化） OR 题目或关键词：（活动监测） OR 题目或关键词：（目标设定）OR 题目或关键词：（活动安排）OR 题目或关键词：（BA疗法）) |

**Table S3 Excluded studies and reasons for exclusion**

| **No.** | **PMID/DOI/…** | **Author** | **Non-T2DM** | **Non-BA** | **Ineligible comparator** | **No available data** | **Duplicate** |
| --- | --- | --- | --- | --- | --- | --- | --- |
| 1 | 10.13342/j.cnki.cjhp.2015.11.006 | Zhang, 2015 | √ |  |  |  |  |
| 2 | 10.1186/s12888-023-04546-w | Abbas, 2023 |  | √ |  |  |  |
| 3 | 10.1177/08901171231224889 | Almutairi, 2024 |  | √ |  |  |  |
| 4 | 10.1186/s13063-024-08171-0 | Bernocchi, 2024 |  | √ |  |  |  |
| 5 | 10.1016/s0277-9536(98)00403-1 | Daniel, 1999 |  | √ |  |  |  |
| 6 | 10.1186/s13063-022-06764-1 | Deverts, 2022 |  | √ |  |  |  |
| 7 | 10.1186/1745-6215-11-35 | Egede, 2010 |  | √ |  |  |  |
| 8 | 10.2196/17968 | Gimbel, 2020 |  | √ |  |  |  |
| 9 | 10.1136/bmjopen-2024-092158 | Glynn, 2025 |  | √ |  |  |  |
| 10 | 10.1016/j.jpsychores.2022.110781 | Gulley, 2022 |  | √ |  |  |  |
| 11 | 10.1016/j.socscimed.2025.117858 | Hawkes, 2025 |  | √ |  |  |  |
| 12 | 10.1093/tbm/ibac014 | Huebschmann, 2022 |  | √ |  |  |  |
| 13 | 10.1111/jnu.12521 | Kim, 2019 |  | √ |  |  |  |
| 14 | 10.1007/s10865-020-00196-5 | Lee, 2021 |  | √ |  |  |  |
| 15 | 10.2337/dc09-2153 | Lorig, 2010 |  | √ |  |  |  |
| 16 | 10.1111/dme.12440 | Maindal, 2014 |  | √ |  |  |  |
| 17 | 10.1177/1403494811402721 | Maindal, 2011 |  | √ |  |  |  |
| 18 | 10.2196/25955 | Martinez, 2021 |  | √ |  |  |  |
| 19 | 10.1186/s13063-019-3712-x | Myers, 2019 |  | √ |  |  |  |
| 20 | 10.1016/j.diabres.2023.110991 | Nelson, 2023 |  | √ |  |  |  |
| 21 | 10.1016/j.erap.2019.03.001 | Pansu, 2019 |  | √ |  |  |  |
| 22 | 10.1016/j.diabres.2023.110921 | Roddy, 2023 |  | √ |  |  |  |
| 23 | 10.5958/0976-5506.2018.01430.4 | Sampath Kumar, 2018 |  | √ |  |  |  |
| 24 | 10.1186/s13063-024-08513-y | Sanders, 2024 |  | √ |  |  |  |
| 25 | 10.1186/s13063-018-2528-4 | Sarfati, 2018 |  | √ |  |  |  |
| 26 | 10.1016/j.cct.2018.10.007 | Shomaker, 2018 |  | √ |  |  |  |
| 27 | 10.1177/0145721710371523 | Wolever, 2010 |  | √ |  |  |  |
| 28 | 10.13342/j.cnki.cjhp.2015.03.038 | Wang, 2015 |  |  |  |  | √ |
| 29 | GRANTS:11328739 | Egede, 2019 |  |  |  |  | √ |
| 30 | 10.1186/s13063-021-05744-1 | Egede, 2021 |  |  |  |  | √ |
| 31 | 10.1186/1472-6963-14-191 | Cully , 2014 | √ |  |  |  |  |
| 32 | 10.1186/s13063-021-05744-1 | Egede, 2021 |  |  |  | √ |  |
| 33 | 10.2337/dc08-0032 | Katon, 2008 | √ |  |  |  |  |
| 34 | 10.1177/1357633X17730419 | Egede, 2018 |  |  | √ |  |  |

**Table S4. Funding and registration information for the included studies**

| **Author/Year** | **Country** | **Registration** | **Find** |
| --- | --- | --- | --- |
| Li 2021 | China | NR | NR |
| Ahmed et al.,2025 | Bangladesh | ISRCTN/75501608 | the National Institute of Health Research, Global Health Research project NIHR200806 |
| Bae et al.,2024 | Korea | NCT05992545 | he Korean Diabetes Association and Sanofi-Aventis Korea |
| Kaltman et al.,2016 | USA | NR | NR |
| Schneider et al.,2016 | USA | NCT01024790 | grant R34 MH086678-01 to Dr. Schneider from the National Institute of Mental Health |
| Vickery et al.,2024 | USA | NCT05258630 | the National Institutes of Health's National Center for Advancing Translational Sciences, Grant UL1TR002494, and the National Institute Of Diabetes And Digestive And Kidney Diseases Grant K23DK118117. |

**Table S5. Outcome measures and data used for meta-analysis**

**1. HbA1c**

| **Study** | **Instrument/measure** | **Time point** | **BA, n** | **BA mean change ± SD** | **Control, n** | **Control mean change ± SD** | **Effect measure** |
| --- | --- | --- | --- | --- | --- | --- | --- |
| Li 2021 | HbA1c (%) | Post-intervention | 11 | -1.12 ± 0.59 | 9 | -0.49 ± 1.87 | MD |
| Ahmed 2025a | HbA1c (%) | 3 months | 17 | -0.20 ± 1.85 | 26 | 0.10 ± 2.29 | MD |
| Ahmed 2025b | HbA1c (%) | 3 months | 29 | -0.20 ± 2.04 | 30 | -0.10 ± 2.31 | MD |
| Bae 2024 | HbA1c (%) | 12 weeks | 20 | 0.00 ± 0.72 | 19 | -0.30 ± 1.76 | MD |
| Schneider 2016 | HbA1c (%) | 6 months | 15 | -0.20 ± 0.72 | 14 | -0.10 ± 0.85 | MD |
| Vickery 2024 | HbA1c (%) | 3 months | 18 | -0.60 ± 2.21 | 18 | -0.80 ± 2.31 | MD |

Negative values indicate improvement. Ahmed 2025a and Ahmed 2025b refer to the Pakistan and Bangladesh subgroups, respectively.

**2. Depression symptoms**

| **Study** | **Instrument/**  **measure** | **Time point** | **BA, n** | **BA mean change ± SD** | **Control, n** | **Control mean change ± SD** | **Effect measure** |
| --- | --- | --- | --- | --- | --- | --- | --- |
| Li 2021 | PHQ-9 | Post-intervention | 11 | -2.27 ± 0.93 | 9 | -0.56 ± 1.72 | Hedges’ g |
| Ahmed 2025a | PHQ-9 | 3 months | 22 | -9.20 ± 5.67 | 29 | -6.50 ± 6.32 | Hedges’ g |
| Ahmed 2025b | PHQ-9 | 3 months | 29 | -9.80 ± 4.83 | 30 | -1.50 ± 5.86 | Hedges’ g |
| Bae 2024 | PHQ-9 | 12 weeks | 20 | -2.40 ± 3.93 | 19 | -1.74 ± 3.66 | Hedges’ g |
| Schneider 2016 | HAMD | 6 months | 15 | -8.20 ± 5.14 | 14 | -5.10 ± 5.51 | Hedges’ g |

SMD, standardized mean difference; PHQ-9, Patient Health Questionnaire-9; HAMD, Hamilton Depression Rating Scale. Negative values indicate improvement. Ahmed 2025a and Ahmed 2025b refer to the Pakistan and Bangladesh subgroups, respectively.

**3. Diabetes self-efficacy**

| **Study** | **Instrument/**  **measure** | **Time point** | **BA, n** | **BA mean change ± SD** | **Control, n** | **Control mean change ± SD** | **Effect measure** |
| --- | --- | --- | --- | --- | --- | --- | --- |
| Ahmed 2025a | DES-SF | 6 months (follow-up results only) | 21 | 0.30 ± 0.72 | 27 | 0.30 ± 0.70 | Hedges’ g |
| Ahmed 2025b | DES-SF | 6 months (follow-up results only) | 30 | 0.20 ± 0.53 | 31 | 0.10 ± 0.50 | Hedges’ g |
| Bae 2024 | DSES | 12 weeks | 20 | -3.20 ± 15.10 | 19 | 0.61 ± 21.69 | Hedges’ g |

SMD, standardized mean difference; DES-SF, Diabetes Empowerment Scale-Short Form; DSES, Diabetes Self-Efficacy Scale. Positive values generally indicate improvement. Ahmed 2025a and Ahmed 2025b refer to the Pakistan and Bangladesh subgroups, respectively.

**4. Diabetes self-management**

| **Study** | **Instrument/**  **measure** | **Time point** | **BA, n** | **BA mean change ± SD** | **Control, n** | **Control mean change ± SD** | **Effect measure** |
| --- | --- | --- | --- | --- | --- | --- | --- |
| Li 2021 | SDSCA-6 | Post-intervention | 11 | 0.83 ± 1.01 | 9 | 0.31 ± 0.61 | Hedges’ g |
| Ahmed 2025a | PDSMS | 6 months (follow-up results only) | 21 | 0.80 ± 3.14 | 27 | 0.60 ± 3.57 | Hedges’ g |
| Ahmed 2025b | PDSMS | 6 months (follow-up results only) | 30 | -1.50 ± 3.76 | 31 | -0.40 ± 3.82 | Hedges’ g |
| Bae 2024 | DSMB-O | 12 weeks | 20 | 3.11 ± 3.78 | 19 | 2.46 ± 4.54 | Hedges’ g |
| Vickery 2024 | DSMQ overall | 3 months | 18 | 0.50 ± 1.20 | 18 | 0.30 ± 1.11 | Hedges’ g |

SMD, standardized mean difference; SDSCA-6, Diabetes Self-Management Behavior Scale-6; PDSMS, Perceived Diabetes Self-Management Scale; DSMB-O, Diabetes Self-Management Behaviors for Older Koreans; DSMQ, Diabetes Self-Management Questionnaire. Positive values generally indicate improvement. Ahmed 2025a and Ahmed 2025b refer to the Pakistan and Bangladesh subgroups, respectively.

**Table S6. Leave-one-out sensitivity analyses**

| **Outcome** | **Study omitted** | **Effect measure** | **Pooled effect estimate** | **95% CI** | ***p*** | **I²** |
| --- | --- | --- | --- | --- | --- | --- |
| Depressive symptoms | Li 2021 | Hedges’ g | -0.75 | -1.21 to -0.29 | 0.001 | 54% |
| Depressive symptoms | Ahmed 2025a | Hedges’ g | -0.77 | -1.30 to -0.23 | 0.005 | 56% |
| Depressive symptoms | Ahmed 2025b | Hedges’ g | -0.69 | -1.14 to -0.24 | 0.003 | 37% |
| Depressive symptoms | Bae 2024 | Hedges’ g | -1.00 | -1.34 to -0.67 | ＜0.00001 | 0% |
| Depressive symptoms | Schneider 2016 | Hedges’ g | -0.87 | -1.36 to -0.37 | 0.0006 | 55% |
| HbA1c | Li 2021 | MD | -0.00 | -0.27 to 0.27 | 0.99 | 0% |
| HbA1c | Ahmed 2025a | MD | -0.02 | -0.31 to 0.27 | 0.90 | 0% |
| HbA1c | Ahmed 2025b | MD | -0.04 | -0.34 to 0.27 | 0.80 | 0% |
| HbA1c | Bae 2024 | MD | -0.09 | -0.38 to 0.19 | 0.52 | 0% |
| HbA1c | Schneider 2016 | MD | -0.03 | -0.31 to 0.25 | 0.84 | 0% |
| HbA1c | Vickery 2024 | MD | -0.06 | -0.35 to 0.22 | 0.66 | 0% |
| Diabetes self-efficacy | Ahmed 2025a | Hedges’ g | 0.04 | -0.35 to 0.43 | 0.85 | 0% |
| Diabetes self-efficacy | Ahmed 2025b | Hedges’ g | -0.09 | -0.51 to 0.33 | 0.68 | 0% |
| Diabetes self-efficacy | Bae 2024 | Hedges’ g | 0.11 | -0.27 to 0.48 | 0.58 | 0% |
| Diabetes self-management | Li 2021 | Hedges’ g | -0.01 | -0.30 to 0.28 | 0.92 | 0% |
| Diabetes self-management | Ahmed 2025a | Hedges’ g | 0.05 | -0.29 to 0.38 | 0.79 | 8% |
| Diabetes self-management | Ahmed 2025b | Hedges’ g | 0.18 | -0.15 to 0.51 | 0.28 | 0% |
| Diabetes self-management | Bae 2024 | Hedges’ g | 0.02 | -0.30 to 0.33 | 0.91 | 4% |
| Diabetes self-management | Vickery 2024 | Hedges’ g | 0.02 | -0.29 to 0.33 | 0.91 | 3% |

MD, mean difference; CI, confidence interval. Leave-one-out sensitivity analyses were conducted by omitting one study or dataset at a time and recalculating the pooled effect estimate using the same random-effects model as the primary analysis.

**Table S7. Egger’s test for publication bias**

| **Outcome** | **Number of datasets** | **Egger’s test statistic** | ***p*** | **Interpretation** |
| --- | --- | --- | --- | --- |
| Depressive symptoms | 5 | z = -0.399 | 0.690 | No significant asymmetry |
| HbA1c | 6 | z = -0.259 | 0.796 | No significant asymmetry |
| Diabetes self-efficacy | 3 | Not performed | — | Too few datasets |
| Diabetes self-management | 5 | z = 1.708 | 0.088 | No significant asymmetry |

**Table S8. GRADE assessment of certainty of evidence**

| **Outcome** | **Studies / participants** | **Effect estimate** | **Risk of bias** | **Inconsistency** | **Indirectness** | **Imprecision** | **Publication bias** | **Certainty of evidence** |
| --- | --- | --- | --- | --- | --- | --- | --- | --- |
| Depressive symptoms | 4 RCTs /  n = 198 | Hedges’ g = -0.81, 95% CI: -1.22 to -0.41 | Serious | Not serious | Not serious | Serious | Not serious | Low |
| HbA1c | 5 RCTs /  n = 226 | MD = -0.04%, 95% CI: -0.30 to 0.22 | Serious | Not serious | Not serious | Serious | Not serious | Low |
| Diabetes self-efficacy | 2 RCTs /  n = 148 | Hedges’ g = 0.03, 95% CI: -0.30 to 0.35 | Serious | Not serious | Not serious | Very serious | Not serious | Very low |
| Diabetes self-management | 4 RCTs /  n = 204 | Hedges’ g = 0.04, 95% CI: -0.23 to 0.32 | Serious | Not serious | Not serious | Serious | Not serious | Low |

Supplementary Figure S1. Exploratory subgroup analysis of depressive symptoms by measurement scale

Supplementary Figure S2. Exploratory subgroup analysis of diabetes self-efficacy by measurement scale

Supplementary Figure S3. Exploratory subgroup analysis of diabetes self-management behaviors by measurement scale

Figure S4. Funnel plot analysis for depressive symptom.

Figure S5. Funnel plot for HbA1c.

Figure S6. Funnel plot for diabetes self-management behaviors.
